# Case Report: Subacute thyroiditis after receiving inactivated SARS-CoV-2 vaccine (BBIBP-CorV)

**DOI:** 10.3389/fmed.2022.918721

**Published:** 2022-07-22

**Authors:** Linhua Pi, Jian Lin, Ying Zheng, Zhen Wang, Zhiguang Zhou

**Affiliations:** ^1^Department of Metabolism and Endocrinology, The Second Xiangya Hospital of Central South University, Changsha, China; ^2^Key Laboratory of Diabetes Immunology (Central South University), Ministry of Education, Changsha, China; ^3^National Clinical Research Center for Metabolic Diseases, Changsha, China; ^4^Center for Medical Research, The Second Xiangya Hospital of Central South University, Changsha, China

**Keywords:** subacute thyroiditis, severe acute respiratory syndrome coronavirus 2, coronavirus disease 2019, vaccine, BBIBP-CorV

## Abstract

**Background:**

Subacute thyroiditis, an inflammatory disease, has been reported caused by vaccines in rare cases. In the context of the coronavirus disease 19 pandemic, various SARS-CoV-2 vaccines have been developed and may be potential triggers for subacute thyroiditis.

**Case presentation:**

We report a case of subacute thyroiditis 3 days after receiving the second dose of inactivated SARS-CoV-2 vaccine (BBIBP-CorV). The patient did not report a previous history of thyroid disease, upper respiratory tract infection, or COVID-19. Physical examination, laboratory testing, ultrasonography, and radioactive iodine uptake were consistent with subacute thyroiditis. During follow-up, the patient recovered from symptoms and signs, and imaging changes except for hypothyroidism, requiring an ongoing thyroxine replacement.

**Conclusions:**

Inactivated SARS-CoV-2 vaccine may be a causal trigger leading to subacute thyroiditis. Clinicians should be aware of subacute thyroiditis as a possible thyroid-related side effect of an inactivated SARS-CoV-2 vaccine.

## Introduction

Subacute thyroiditis is an inflammatory thyroid disease with viral infections as the most common causes. Women are more susceptible than the men. History of preceding upper respiratory tract infection, anterior neck pain, and three-phase changes of thyroid function are the typical characteristics in subacute thyroiditis. Consistent clinical and imaging findings are helpful to make the diagnosis (1).

Rare cases of subacute thyroiditis have also been reported following vaccination, such as influenza (2), hepatitis B vaccine (3), and so on. In the context of the COVID-19 pandemic, urgent demand for effective vaccines against SARS-CoV-2 led to rapid development of SARS-CoV-2 vaccines. Various SARS-CoV-2 vaccines have been developed based on different platforms and proven effective against SARS-CoV-2 (4–8). Despite that a series of subacute thyroiditis cases have been reported after receiving various SARS-CoV-2 vaccines in distinct populations, referring to mRNA vaccine (Pfizer/BioNTech and Modena), adenovirus-vectored vaccine (ChAdOx1 nCoV-19 vaccine), and inactivated vaccine (CoronaVac®), there are no cases of subacute thyroiditis following the administration of an inactivated SARS-CoV-2 vaccine (BBIBP-CorV) reported in Chinese population yet (9–12).

Here, we present a novel case of subacute thyroiditis following the administration of inactivated SARS-CoV-2 vaccine (BBIBP-CorV, Beijing Institute of Biological Products, China), providing evidence for BBIBP-CorV as a causal trigger for subacute thyroiditis.

## Case presentation

A 32-year-old female patient was admitted to our endocrinology outpatient department because of anterior neck pain and swelling for 26 days. She further complained about receiving the second dose of inactivated SARS-CoV-2 vaccine (BBIBP-CorV) on 19 February 2021, 3 days before the symptoms above started. She did not report any history of recent upper respiratory tract infection or COVID-19. However, she had been prescribed methimazole for 10 days, on account of occasional palpitation due to thyrotoxicosis at the local hospital.

**Figure 1:**
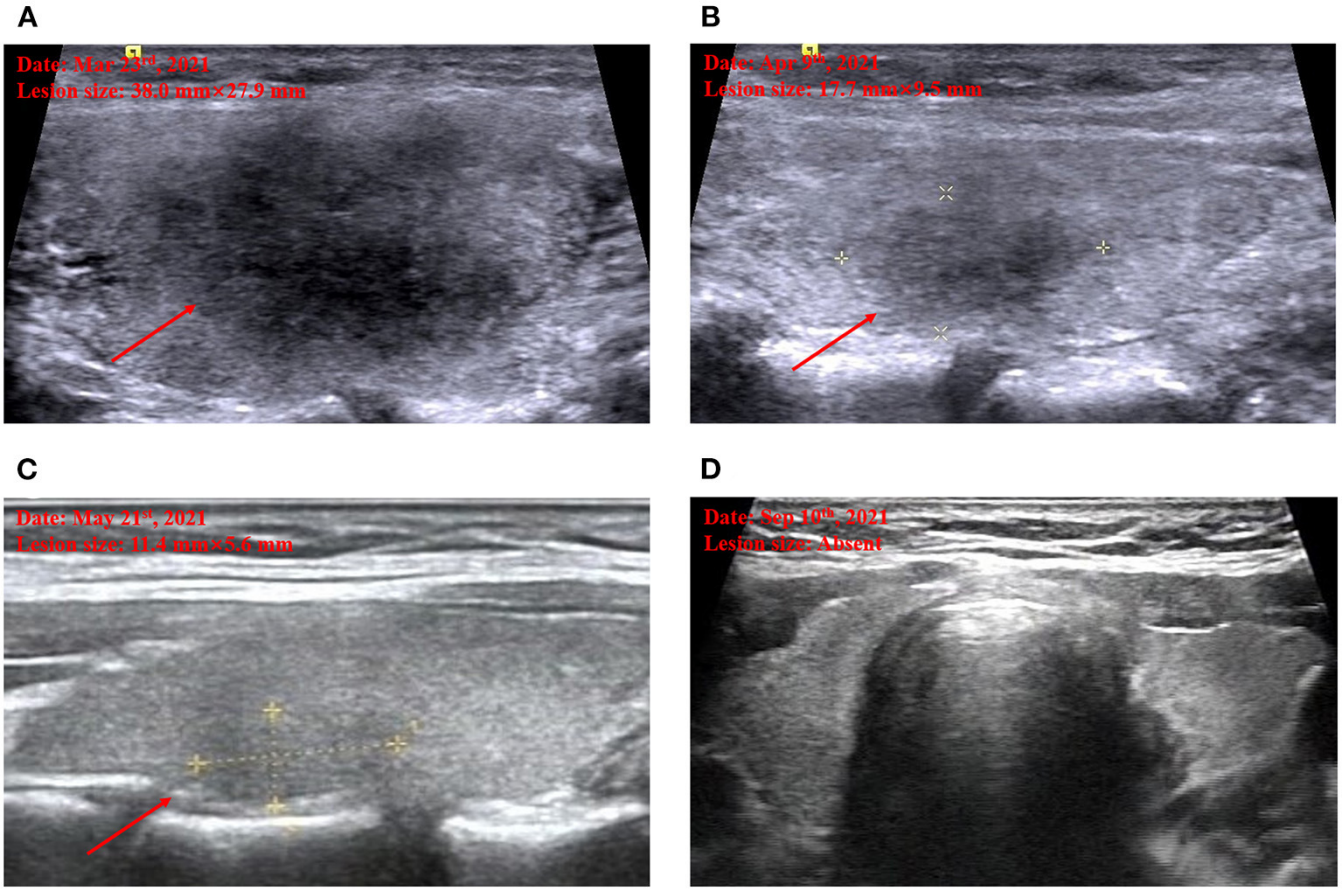
Thyroid ultrasound imaging. Marked hypoechoic lesion on admission **(A)**. Decreased hypoechoic lesion by half when the first follow-up on April 9 **(B)**. Further decreased hypoechoic lesion when the second follow-up on May 21 **(C)**. Disappeared hypoechoic lesion when the third follow-up on September 10 **(D)**.

Upon physical examination, her heart rate was 110/min and body temperature was 36.7°C. On palpation, the thyroid was enlarged and two symmetrical and painful nodules were found about 30 mm in diameter in the bilateral thyroid lobes. Further ultrasound defined the characteristics of the nodules as hypoechoic areas with blurred margins, irregular shapes, and decreased vascularity, in line with the clinical location on physical examination (Figure 1A).

Upon laboratory assessment, the thyroid function showed elevated free triiodothyronine (FT3) and free thyroxine (FT4) and reduced thyrotropin (TSH). Thyroid-related autoantibody detection showed positive thyroglobulin antibodies (TgAb) and thyroid peroxidase autoantibodies (TPOAb) and negative TSH receptor antibodies (TRAb). Inflammatory markers showed normal complete blood count (CBC), significantly elevated erythrocyte sedimentation (ESR), and C-reactive protein (CRP) (Table 1).

**Table 1:** Clinical, laboratory, imaging findings on admission and during follow-up.

**Items**	**Before admission** **(Mar. 10)**	**Reference range**	**Admission** **(Mar. 20)**	**Follow-up** **(Apr. 9)**	**Follow-up** **(May. 21)**	**Follow-up** **(Sep. 10)**	**Reference range**
Symptoms	Neck pain		Neck pain	Relieved	Absent	Absent	
FT3	7.27	2.0–4.2 pg/mL	8.21	3.85	4.09	4.67	3.5–6.5 pmol/L
FT4	28.49	8.9–17.2 pg/mL	22.87	8.75	15.21	15.92	11.5–22.7 pmol/L
TSH	0.035	0.3–4.5 uIU/mL	<0.01	2.18	3.98	3.96	0.55–4.78 uIU/mL
TgAb			257.4				0–60 IU/mL
TPOAb	62.56	≤34 IU/mL	538.8				0–60 IU/mL
TRAb			0.3				<1.5 IU/mL
WBC	4,520	4,000–10,000 per L	6,440	6,260	7,300	6,300	3,500–9,500 per L
ESR	68	0–20 mm/h	57	14	14	7	0–15 mm/h
CRP	69.58	0–10 mg/L	20	2.3			0–6 mg/L
RAIU			Reduced				
Hypoechoic area	L: 35 × 30 mm R: 26 × 24 mm		L: 38 × 28 mm R: 26 × 22 mm	L: 18 × 10 mm R: 15 × 15 mm	L: 11 × 5 mm R: 10 × 6 mm	Absent	
Treatment	MMI		Celecoxib Propranolol	Levothyroxine	Levothyroxine	Levothyroxine	

FT3, free triiodothyronine; FT4, free thyroxine; TgAb, thyroglobulin antibodies; TPOAb, thyroperoxidase antibodies; TRAb, TSH receptor antibodies; TSH, thyrotropin; WBC, white blood cell; CRP, C reactive protein; ESR, erythrocyte sedimentation rate; RAIU, radioactive iodine uptake; MMI, methimazole.

**Figure 2:**
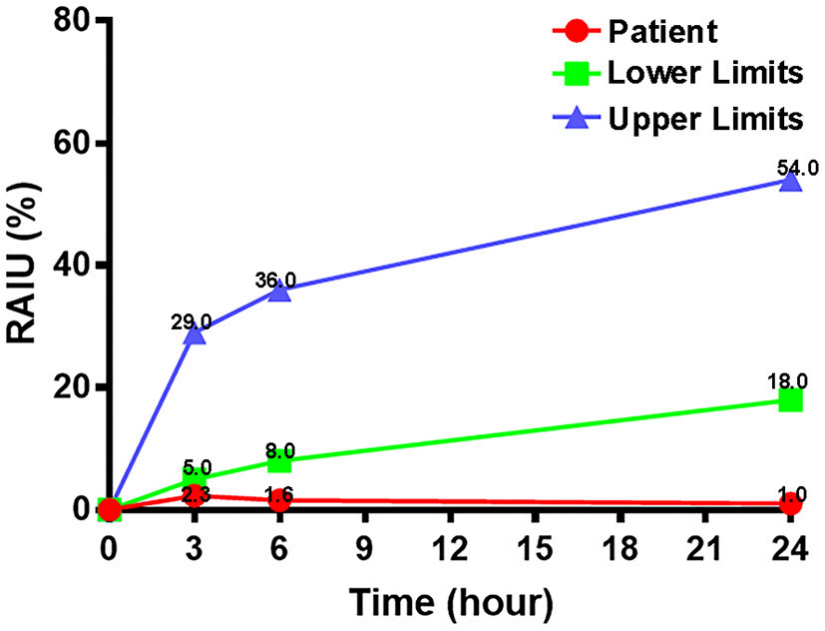
Thyroid radioactive iodine uptake.

In order to differentiate the etiology of thyrotoxicosis, a radioactive iodine uptake (RAIU) was performed and found to be significantly low (Figure 2). The separation phenomenon of reduced RAIU and elevated thyroid hormones implied a destructive cause of thyrotoxicosis in this patient.

According to susceptible HLA genotypes reported in subacute thyroiditis (13), we performed an HLA genotyping and found that the genotypes are HLA-B^*^46:01/58:01, HLA-C^*^01:02/03:02, and HLA-DRB1^*^03:01/09:01 in the current case.

Based on all findings above, the diagnosis of subacute thyroiditis was confirmed and the medications were switched from methimazole 10 mg daily started in the local hospital to celecoxib 200 mg daily and propranolol 10 mg three times a day in our outpatient department, to control the inflammation and thyrotoxicosis-related symptoms. During the follow-up, neck pain was relieved 20 days later; hypoechoic areas were decreased gradually and disappeared 6 months later (Figures 1B–D). The thyroid function turned out to hypothyroidism with gradually decreasing free T4 level to below the lower limit of normal range 20 days later after withdrawing methimazole, despite the normal free T3 and TSH level, and requiring an ongoing levothyroxine 50 ug daily even 6 months later, to maintain a normal thyroid function. Long-term follow-up would be conducted to determine transient or permanent hypothyroidism in the current case, and whether or not the patient would need levothyroxine treatment constantly.

**Figure 3:**
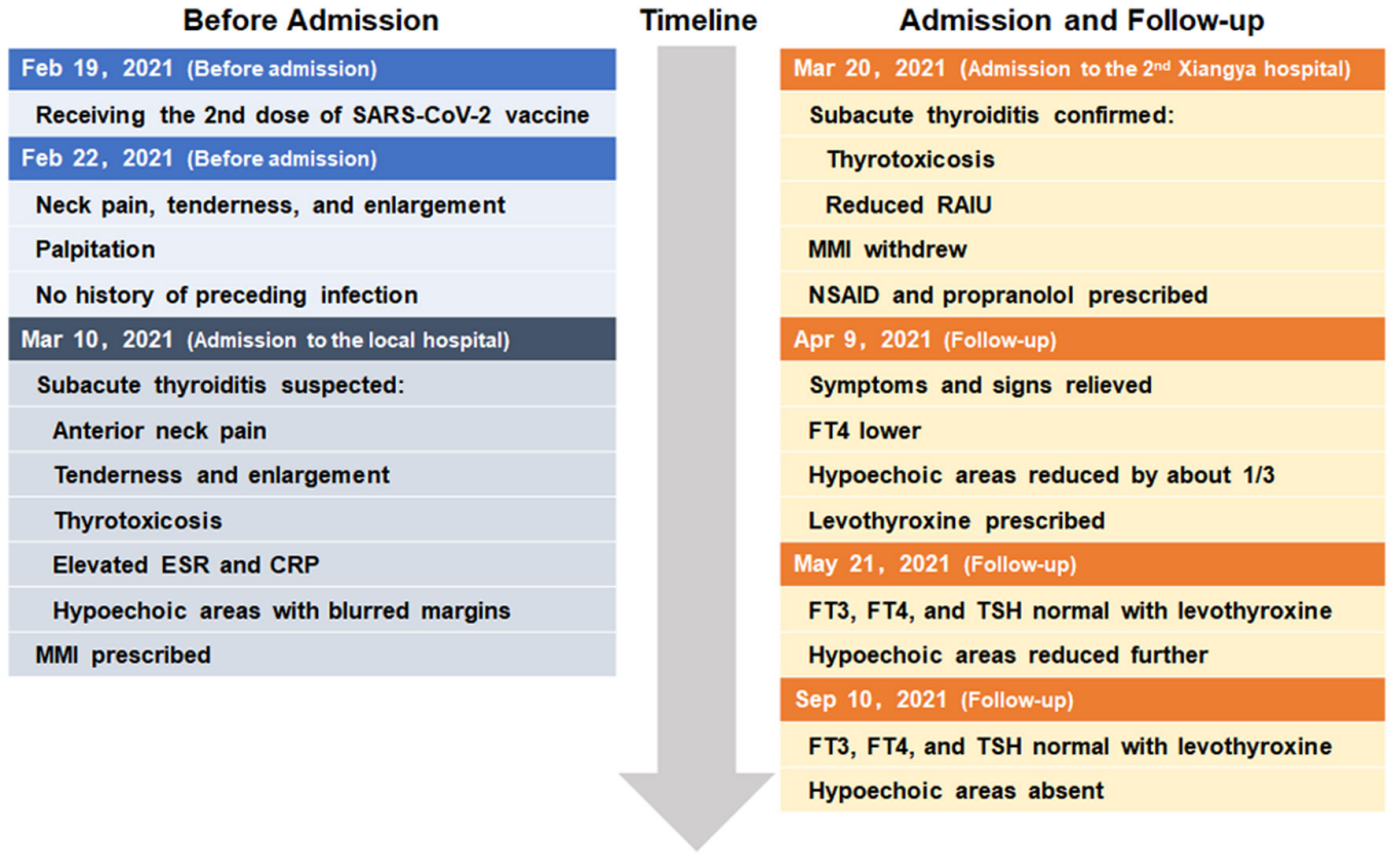
The whole timeline of clinical progression.

The whole timeline of clinical progression is shown in Figure 3. Clinical, laboratory, imaging findings on admission and during follow-up are shown in Table 1.

## Discussion

Subacute thyroiditis, also known as De Quervain's thyroiditis or granulomatous thyroiditis, is the most common form of painful thyroiditis. It usually develops with a preceding history of upper respiratory tract infection and most often occurs at the age of 40–50 in women. The diagnostic clues include neck pain, early thyrotoxicosis condition, typical ultrasound changes with consistent clinical location on physical examination, and elevated inflammatory markers (1).

Viral infections are the most common causes of subacute thyroiditis. In the context of COVID-19 pandemic, SARS-CoV-2 has been considered a causal trigger for subacute thyroiditis (14). In addition, rare cases of subacute thyroiditis have also been reported following vaccination against viruses, such as influenza (2), hepatitis B vaccine (3), and so on. In the current case, the patient developed neck pain 3 days after the second dose of inactivated SARS-CoV-2 vaccine (BBIBP-CorV). In addition, the patient did not have history of thyroid disease or recent history of virus infection or COVID-19. Thus, it was speculated that the development of subacute thyroiditis in this case was triggered by the administration of BBIBP-CorV.

SARS-CoV-2 vaccination was the most important strategy to curb the COVID-19 pandemic (15). Based on different platforms, inactivated vaccines, RNA vaccines, and adenovirus-vectored vaccines were developed and all of them were reported effective in producing neutralizing antibodies (5–8, 16). However, some cases of inflammatory thyroid disease were also reported occurring after receiving SARS-CoV-2 vaccines. Iremli et al. from Turkey reported three cases of subacute thyroiditis following the administration of inactivated SARS-CoV-2 vaccine (CoronaVac®, Sinovac Life Sciences, China) (12). Franquemont and Galvez reported that subacute thyroiditis occurred 5 days after the first dose of mRNA vaccine (Comirnaty vaccine, Pfizer/BioNTech) for COVID-19 (10). Oyibo reported 55-year-old woman developed subacute thyroiditis 3 weeks after receiving the first dose of adenovirus-vectored SARS-CoV-2 vaccine (ChAdOx1 nCoV-19 vaccine, AstraZeneca) (11). Various SAR-CoV-2 vaccines seemed to be related to subacute thyroiditis. Herein, we firstly presented a Chinese case, who developed subacute thyroiditis after receiving another inactivated SARS-CoV-2 vaccine candidate, BBIBP-CorV. BBIBP-CorV is an inactivated SARS-CoV-2 vaccine candidate, which was proven to be safe and well tolerated (5). However, the mechanisms of the SARS-CoV-2-vaccine-induced subacute thyroiditis remained unclear.

As there are many proteins originated from or similar to the pathogen virus in the inactivated virus vaccines, molecular mimicry seems to play a potential role in the development of inactivated SARS-CoV-2-vaccine-related subacute thyroiditis. Spike (S) protein is an immunodominant antigen of SARS-CoV-2, which is also the target of neutralizing antibodies (8, 15, 17). A previous study has proven that there is similarity between S protein and various self-tissue proteins in human (18). It has also been shown that thyroid peroxidase (TPO) sequences share homology and similarity with sequences in S protein, which to extent supported another finding that human monoclonal antibodies against SARS-CoV-2 S protein react with TPO (19). Furthermore, by comparing immunogenic epitopes of SARS-CoV-2 with human proteins, Lyons-Weiler found a high degree of homology with various tissues including thyroid gland (20). In addition, a pilot study further implied that proinflammatory cytokines could increase the expression of angiotensin-converting-enzyme-2 (ACE-2), the receptor for cellular entry of SARS-CoV-2, which might facilitate the entering of SARS-CoV-2 into thyroid and contribute to the understanding of pathogenesis in subacute thyroiditis after SARS-CoV-2 infection or vaccination (21).

Aluminum hydroxide, traditionally used as vaccine adjuvant compound, can enhance immune response and enable the usage of smaller amount of antigens in vaccines (22, 23). Bragazzi et al. have proposed that adjuvant might be associated with the onset of subacute thyroiditis, and they also defined the term subacute autoimmune thyroiditis (24). However, there is a lack of direct evidence supporting the hypothesis of autoimmune syndromes induced by adjuvants (ASIA) in subacute thyroiditis, which has also been well discussed in case series of vaccine-associated subacute thyroiditis by Yorulmaz et al. (25). Furthermore, through the subacute thyroiditis cases reported with non-adjuvanted SARS-CoV-2 vaccines (BNT162b2, Moderna), it has become more evident that SARS-CoV-2 vaccine itself (mainly S protein) plays a more important role than adjuvant in the development of subacute thyroiditis (9, 10). In subacute thyroiditis cases related with inactivated SARS-CoV-2 vaccines, however, the role and mechanism of adjuvant remain to be elucidated in the future.

Certain types of human leukocyte antigens (HLA) have been considered to account for susceptibility to subacute thyroiditis (1). In addition to previously well-known HLA-B^*^35, subacute thyroiditis was proven to be associated with the presence of HLA-B^*^18:01-DRB1^*^01 and -C^*^04:01, which could predispose to subacute thyroiditis and be associated with ultrasound patterns and prognosis of subacute thyroiditis (1, 13). In the current case, however, we found that the HLA genotypes were HLA-B^*^46:01/58:01, HLA-C^*^01:02/03:02, and HLA-DRB1^*^03:01/09:01. We speculated that there might be some other susceptible HLA genotypes involved with the risk of subacute thyroiditis following inactivated SARS-CoV-2 vaccination. Moreover, further studies are needed to verify the association between our findings of HLA genotypes with the risk of subacute thyroiditis following inactivated SARS-CoV-2 vaccination in a large population.

When it was difficult to confirm the diagnosis of subacute thyroiditis, histological workup could be helpful to diagnosis and differential diagnosis, which could also to extent imply possible pathogenesis on histological level (1). After reviewing subacute thyroiditis cases related to SARS-CoV-2 vaccination published in PUBMED database, we found a few cases with a brief histological description as showing lymphocytic infiltrates, macrophages, multinucleated giant cells, and granulomatous inflammation, which were consistent with subacute thyroiditis (9, 26–28). Despite the different causes from classical forms, subacute thyroiditis triggered by various SARS-CoV-2 vaccines showed similar characteristics in histological investigation.

At present, almost all researchers focused mainly on non-specific adverse events for SARS-CoV-2 vaccines (29), but less attention was placed on specific diseases, which were presented only in rare case reports, such as subacute thyroiditis (11, 30, 31), hypophysitis (32), autoimmune hepatitis (33), granulomatous vasculitis (34), autoimmune thrombocytopenic purpura (35), myocarditis (36), and so on. However, in the current context of scale-up of mass vaccination against COVID-19, as well as the efficacy, adverse events should be monitored continuously to enhance awareness of autoimmune or inflammatory conditions such as subacute thyroiditis following SARS-CoV-2 vaccination.

## Conclusion

Herein, we firstly reported a Chinese case developing subacute thyroiditis after receiving BBIBP-CorV, an inactivated SARS-CoV-2 vaccine. The mechanism of subacute thyroiditis triggered by SARS-CoV-2 vaccines is not clear. Further attention should be needed to pay to the development of subacute thyroiditis after receiving SARS-CoV-2 vaccines in Chinese population. Additionally, studies are needed to better understand the incidence and underlying mechanisms of SARS-CoV-2-vaccine-associated subacute thyroiditis.

## Data availability statement

The raw data supporting the conclusions of this article will be made available by the authors, without undue reservation.

## Ethics statement

The studies involving human participants were reviewed and approved by the Ethics Committee of the Second Xiangya Hospital, Central South University. The patients/participants provided their written informed consent to participate in this study. Written informed consent has been obtained from the patient for publication of the case report and accompanying images.

## Author contributions

LP and JL collected the case and data. ZW wrote the manuscript. YZ and ZZ contributed to discussion. All authors contributed to the article and approved the submitted version.

## Conflict of interest

The authors declare that the research was conducted in the absence of any commercial or financial relationships that could be construed as a potential conflict of interest.

## Publisher's note

All claims expressed in this article are solely those of the authors and do not necessarily represent those of their affiliated organizations, or those of the publisher, the editors and the reviewers. Any product that may be evaluated in this article, or claim that may be made by its manufacturer, is not guaranteed or endorsed by the publisher.
